# Group-assisted purification (GAP) chemistry for the synthesis of Velcade via asymmetric borylation of *N*-phosphinylimines

**DOI:** 10.3762/bjoc.10.69

**Published:** 2014-03-31

**Authors:** Jian-bo Xie, Jian Luo, Timothy R Winn, David B Cordes, Guigen Li

**Affiliations:** 1 Department of Chemistry and Biochemistry, Texas Tech University, Lubbock, Texas 79409-1061, United States; 2 Institute of Chemistry & BioMedical Sciences (ICBMS), Nanjing University, Nanjing 210093, P. R. China

**Keywords:** asymmetric borylation, GAP chemistry, organophosphorous, *N*-phosphinylimine, Velcade

## Abstract

A new approach to the anticancer drug Velcade was developed by performing asymmetric borylation of an imine anchored with a chiral *N*-phosphinyl auxiliary. Throughout the 7-step synthesis, especially in the imine’s synthesis and in the asymmetric borylation reactions, operations and work-up were conducted in simple and easy ways without any column chromatographic purification, which defines the GAP (group-assisted purification) chemistry concept. It was found that the optically pure isomer (dr > 99:1) can be readily obtained by washing the crude mixture of the asymmetric borylation reaction with hexane; the chiral *N*-phosphinyl auxiliary can be easily recovered after deprotection is finished. Several other *N*-phosphinylimines were also investigated for the asymmetric borylation reaction. The absolute configuration of the borylation product was confirmed by single crystal X-ray diffraction analysis.

## Introduction

**Scheme 1:**
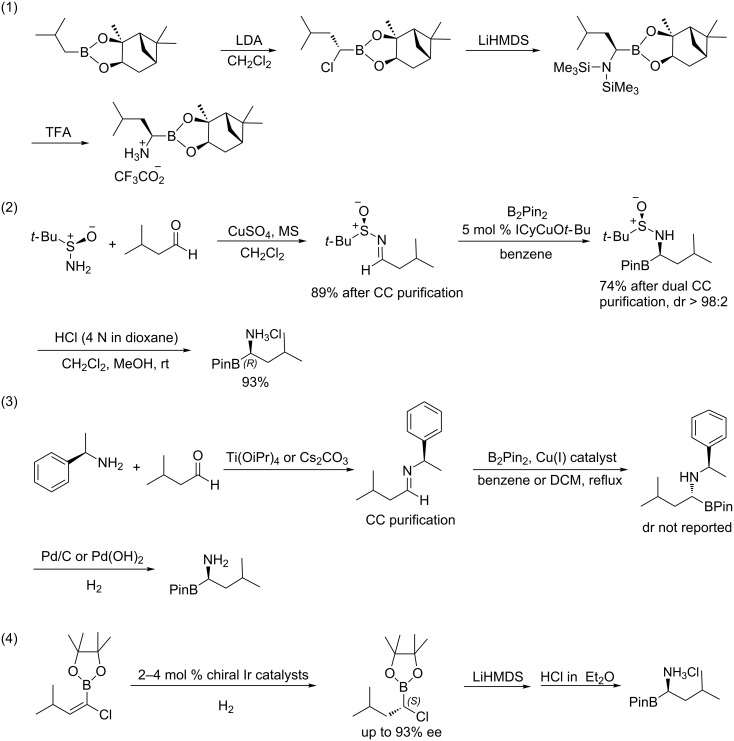
Previous work for (*R*)-(1-amino-3-methylbutyl)boronic acid synthesis.

The synthesis of chiral α-aminoboronic acids and their derivatives has attracted much attention in the organic and medicinal chemistry communities because of their importance for drug discovery and biological research [1–8]. In the past several years, asymmetric catalysis and auxiliary-directed asymmetric synthesis has been conducted for assembling adjacent chiral centers of boronic aicds [9–13], but only a few methods for generating chiral α-aminoboronic acids have been reported so far [14–20]. Among the resulting products from the above methods, (*R*)-(1-amino-3-methylbutyl)boronic acid served as the key mechanism-based pharmacophore in the anticancer drug Velcade, which was the first FDA approved proteasome inhibitor, and has been in clinical use for the treatment of multiple myeloma and mantle cell lymphoma [21]. This product is usually synthesized in three representative processes: (1) to use (1*S*,2*S*,3*R*,5*S*)-(+)-2,3-pinanediol as the chiral auxiliary for the addition reaction followed by chlorination and amination [14–15]; (2) to perform copper-catalyzed borylation of the imine anchored with chiral auxiliaries [16–17]; (3) to conduct asymmetric catalytic hydrogenation as the key step to control the chiral center of boronic aicd [18]. As anticipated, the above known syntheses required traditional purification methods using column chromatography or recrystallization (Scheme 1).

Recently, our group has established a concept called GAP (group-assisted purification) chemistry for greener synthesis [22–25]. This concept describes a process where special functional groups are attached onto reaction substrates, facilitating purification of crude products by avoiding traditional purification methods such as chromatography or recrystallization. The pure products, often pure stereoisomers, can be easily obtained by washing the crude products with common solvents or co-solvents [22–25]. Our GAP concept was attributed to the study of a series of new compounds containing achiral/chiral *N*-phosphonyl and chiral *N*-phosphinylimines, and the reactions of these compounds.

The requirements of GAP chemistry are shown by the fact that the functional groups of the reactants should generate products of adequate solubility. The GAP products should be soluble in some solvents such as THF and DCM for further reactions. However, they should have poor solubility in other solvents such as petroleum ether, hexanes, and their co-solvents with EtOAc. The GAP requirements should include adequate chemical reactivity of GAP compounds towards many reactants and species. If GAP groups are chiral, they should control asymmetric additions efficiently. Our GAP functional groups have also showed the flexibility for structural modifications in order to control the solubility of products and also to control the chemical tolerance towards various reactions under different conditions. Moreover, the GAP auxiliaries have been proven to be easily deprotected under several conditions for re-use.

**Scheme 2:**
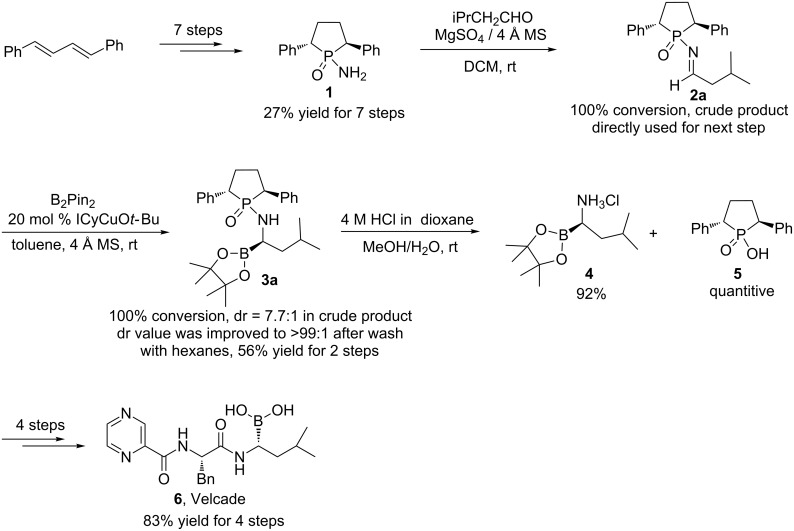
Synthesis of (*R*)-**4** and Velcade.

Herein, we report the synthesis of the anticancer drug Velcade and its derivatives of chiral α-aminoboronic esters via GAP chemistry. Simple operations are needed during purification without the need for column chromatography; GAP washing can lead to a single isomeric product, which was deprotected with quantitative recovery of the phosphinic acid as shown in Scheme 2.

## Results and Discussion

We started our synthesis with (2*S*,5*S*)-1-amino-2,5-diphenylphospholane 1-oxide (**1**), which was synthesized according to the literature and our previous work in 7 steps from (1*E*,3*E*)-1,4-diphenylbuta-1,3-diene with an overall high yield (27% for 7 steps) [25–26]. With the optically pure amide **1** in hand, we screened the condensation conditions to generate the imine **2a**. Titanates, such as Ti(OiPr)_4_ and Ti(OEt)_4_, resulted in the complete conversion of the aliphatic aldehyde in two days, but the hemiaminal was obtained as the main product [27], which can slowly decompose to release the desired imine **2a**. Anhydrous CuSO_4_, which was used as a mild condensation reagent by Ellman’s group, was found to be not suitable for this condensation reaction. This lack of suitability can probably be attributed to the strong coordination tendency of the imine to a copper ionic center. TiCl_4_ was proven to be an efficient reagent and lead to complete condensation within 2 hours to give 90% conversion to imine according to crude ^31^P NMR. We also found that when the crude imine product was purified by column chromatography, isomerization of **2a** to enamine was observed; this observation became more obvious when the solvents used for chromatography contained a higher water content. Eventually, MgSO_4_ or CaSO_4_ was chosen as the dehydration reagent together with molecular sieves for imine formation. Under these conditions, complete conversion can be reached after 5 days to give the chiral *N*-phosphinylimine, which can be directly used for the following asymmetric borylation reaction without further purification.

**Figure 1:**
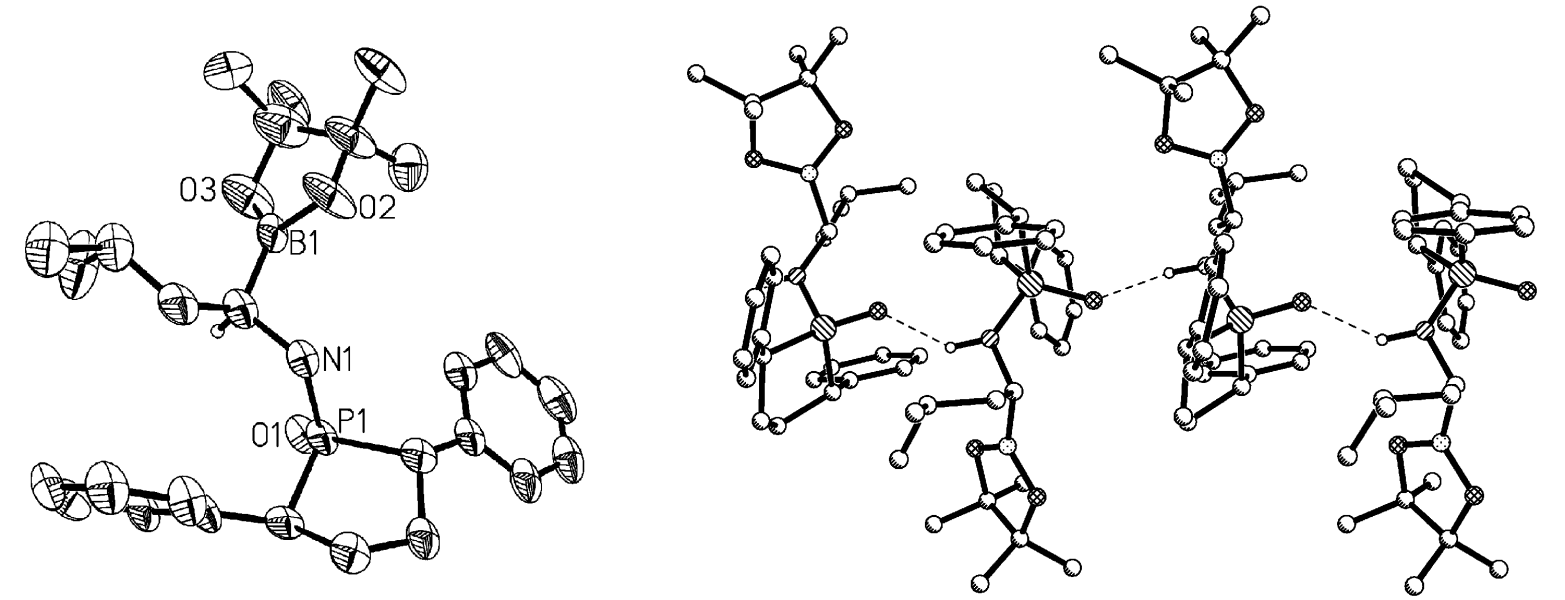
ORTEP diagram of (*S*,*S*,*R*)-**3a** (left, most of the hydrogen atoms were omitted except the one on the chiral center connected to boron) and the H-bonds between the molecules (right). Selected bond lengths [Å] and angle [^o^]: P1–O1 1.505, P1–N1 1.613, N1–H1, 0.957, B1–O2 1.368, the H-bond O1···H2 1.990; the angle of H bond N1–H1···O4 142.79.

B_2_Pin_2_ and ICyCuO*t-*Bu were utilized as the electrophilic addition reagent and catalyst, respectively, for the borylation reaction as previously reported [16,28]. Unlike the previous system, in which benzene was employed as the solvent, we used the much less toxic solvent toluene to successfully replace benzene. We achieved full conversion with 2 equiv of B_2_Pin_2_ in the presence of 20 mol % of ICyCuO*t*-Bu catalyst after 3 days to afford a dr value of 7.7:1 as revealed by crude ^31^P NMR analysis. After simple work-up and washing with hexanes, the dr value was improved to a single isomer (Scheme 2) in 56% yield for two steps. The absolute configuration of product **3a** was unambiguously determined by X-ray analysis [29] to be in the (*S*,*S*,*R*)-configuration (Figure 1). As shown in Figure 1, H-bonds exist between the molecules, which would further help the formation of the solid.

To hydrolyze the resulting aminoboronic ester, the known method [16] was followed first by treating the ester with one equivalent of HCl in co-solvents of methanol and dioxane. A mixture of products was generated indicating a poor yield according to the crude ^31^P and ^1^H NMR analysis. Another co-solvent system consisting of H_2_O and MeOH (2:1) was examined. It was found that treatment of the aminoboronic ester with 1.5 equiv of HCl for 16 hours resulted in complete deprotection; work-up consisted of extraction with DCM followed by concentration to give product **4** as a white solid in 92% yield. Pure phosphinic acid **5** was recovered by simple filtration in quantitative yield. The final product, Velcade **6**, was synthesized according to the literature procedure [16] in 83% yield after 4 steps (Scheme 2). ^1^H NMR and ^13^C NMR spectra of the product were proven to be identical to the literature data.

**Scheme 3:**
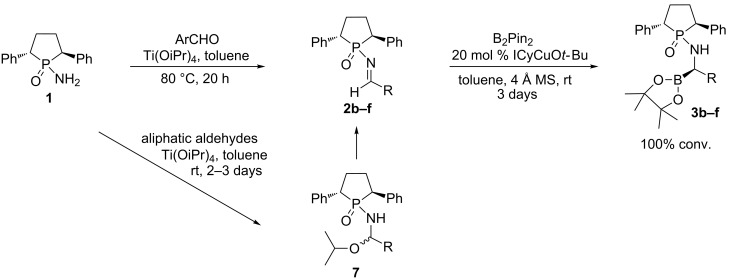
Synthesis of phosphinylimines and their borylation products.

To expand the substrate scope for the borylation reaction, several other new *N*-phosphinyl imines were synthesized for examination (Scheme 3). Ti(OiPr)_4_ was chosen as the general condensation reagent. When aliphatic aldehydes were subjected to the condensation reaction, the main products were generated either as hemiaminals **7** or as a mixture of imine and hemiaminal after purification by column chromatography. The hemiaminals were slowly transferred to the corresponding *N*-phosphinylimine as revealed by NMR analysis. The aliphatic imines are found to be unstable even in the presence of a trace amount of moisture, and can easily isomerize to form the enamine. Therefore, we directly subjected the mixture of imine and hemiaminal to the borylation reaction. The borylation reactions went smoothly with full conversion after stirring the reaction mixture at room temperature for 3 days. Although moderate diastereoselectivities were obtained for all the three aliphatic *N*-phosphinylimines, the diastereopurities can be easily improved by GAP washing with hexane. For example, the original dr value for **3b** of 75:25 can be enhanced to a single isomer (dr > 99:1) in 26% yield over two steps (calculated from amide **1**). When aromatic aldehydes were employed, the yields of *N*-phosphinylimine formations were quantitative; the crude products were almost pure according to crude ^31^P NMR spectra. After simple work-up to remove most of the titanate, the crude products were used directly for the next step without further purification. For the aromatic cases (entries 4 and 5, Table 1), excellent diastereoselectivities, dr = 97:3 and >99:1, respectively, were achieved according to the ^31^P NMR analysis of crude products. Pure products **3e** and **3f** were obtained via flash column chromatography since GAP washing led to some decomposition in these two cases.

**Table 1:** Results of borylation reactions^a^.

Entry	Imines	R	Products	dr (crude)^b^	dr (GAP)^b^	Yield^c^

1	**2b**	iPr	**3b**	75:25	>99:1	26%
2	**2c**	cyclohexyl	**3c**	78:22	99:1	40%
3	**2d**	PhCH_2_CH_2_	**3d**	84:16	98:2	45%
4	**2e**	Ph	**3e**	97:3	–	51%
5	**2f**	4-MeOPh	**3f**	>99:1	–	44%

^a^Reaction conditions: 0.74 mmol scale, [substrate] = 1.8 M, 2.0 equiv B_2_Pin_2_, 20 mol % ICyCuO*t*-Bu, toluene (4 mL), room temperature, 3 days. ^b^Determined by ^31^P NMR analysis. ^c^Calculated from the starting amide **1**, isolated yields with GAP washing (for **3e** and **3f**, GAP washing was not conducted).

## Conclusion

We have successfully demonstrated that *N*-phosphinylimines can undergo electrophilic borylation reactions, and that this reaction can be applied in the synthesis of the anticancer drug Velcade. The *N*-phosphinyl auxiliary displayed good to excellent asymmetric induction and great stability in the catalytic borylation and deprotection reactions. GAP washing is found to enhance the diastereopurity of the borylation products in most cases. The absolute configuration of the borylation product in Velcade’s synthesis has been confirmed by single crystal X-ray diffraction analysis.

## Experimental

Standard operations for catalytic borylation and GAP: A 10 mL Schlenk tube was charged with crude imine **2**, B_2_Pin_2_ (375 mg, 2 equiv), catalyst ICyCuO*t*-Bu (54 mg, 20 mol %) and 4 Å molecular sieves (~500 mg). The mixture was protected with argon atmosphere, followed by toluene (4 mL) addition via syringe. The reaction mixture was stirred vigorously for 3 days, and the resulting slurry was filtered through Celite and washed with EtOAc 5 times. The filtrate was then concentrated and checked by ^31^P NMR to determine the % conversion and the crude dr value. GAP operations: 1) **Method A**: The filtrate was re-dissolved in EtOAc and washed with 1 N HCl, water, and brine successively, then dried over anhydrous Na_2_SO_4_. The crude product was obtained by filtration and concentration, followed by addition of 5 mL of hexanes to triturate the crude product. After 30 minutes, the solvent was decanted and another 5 mL hexanes were added. The resulting slurry was then filtered and the solid was washed with another 2 mL hexanes. The solid product was collected and dried in vacuo. The yield was calculated and the dr value and purity were checked by ^31^P NMR and ^1^H NMR. 2) **Method B**: The filtrate was re-dissolved in EtOAc/hexanes (20 mL, v/v = 1:1), and then filtered through Celite. The filtrate was concentrated and triturated with hexanes (5 mL). After 30 minutes, the solvent was decanted and another 5 mL hexanes was added. The resulting slurry was then filtered and the solid was washed with another 2 mL hexanes. The solid product was collected and dried in vacuo. The yield was calculated from the starting amide **1**. The dr value and purity were checked by ^31^P NMR and ^1^H NMR.

## Supporting Information

File 1 Experimental details, characterization data of all products, and copies of NMR spectra.
