# Supplementary material for: Group-assisted purification (GAP) chemistry for the synthesis of Velcade via asymmetric borylation of N-phosphinylimines
Source: Beilstein J Org Chem. 2014 Mar 31;10:746–51. doi: 10.3762/bjoc.10.69 (PMC3999766; doi:10.3762/bjoc.10.69)
Supplement: File 1 — Experimental details, characterization data of all products, and copies of NMR spectra. [file Beilstein_J_Org_Chem-10-746-s001.pdf]

Supporting Information

for

Group-assisted purification (GAP) chemistry for the synthesis of Velcade via asymmetric borylation of *N*-phosphinylimines

Jian-bo Xie[1], Jian Luo[1], Timothy R. Winn[1], David B. Cordes[1] and Guigen Li[1, 2, *]

Address: [1]Department of Chemistry and Biochemistry, Texas Tech University, Lubbock, Texas 79409-1061, United States and [2]Institute of Chemistry & BioMedical Sciences (ICBMS), Nanjing University, Nanjing 210093, P. R. China

Email: Guigen Li - guigen.li@ttu.edu

* Corresponding author

Experimental details, characterization data of all products, and copies of NMR spectra.

General remarks

All commercially available solvents, unless otherwise mentioned, were used without purification. DCM was distilled from CaH_2. Toluene was distilled from Na/benzophenone. B_2Pin_2 was purchased from Frontier Scientific. CuCl (>99.99%) was purchased from Aldirich. All the glasswares used were dried overnight at 110 °C.

All melting points are uncorrected. The NMR spectra were recorded at 400, 100, and 162 MHz for ^{1}H, ^{13}C, and ^{31}P, respectively with a JEOL ECS 400 MHz spectrometer. Chemical shifts were reported in ppm downfield from internal Me_4Si and external 85% H_3PO_4, respectively. Optical rotations were determined using an Autopol® IV automatic polarimeter. X-ray diffraction analysis was performed on a Bruker smart-1000 X-ray diffraction meter.

Preparation of achiral copper(I) catalyst ICyCuO*t*-Bu

[1,3-Dicyclohexylimidazol-2-ylidene]copper(I) *tert*-butoxide, ICyCuO^{t}Bu, was prepared starting from 1,3-dicyclohexyl-1*H*-imidazol-3-ium chloride (ICy·HCl) [1] according to literature method[2] with slight improvement in one step.

A 10 ml Schlenk tube with a Teflon-coated magnetic stirbar was charged with CuCl (0.41 g, 4.1 mmol), NaO*t*-Bu (0.99 g, 10.3 mmol), and ICy·HCl (1.08 g, 4.0 mmol). The mixture was protected with argon. THF (10 mL) was added, and the reaction mixture was stirred for 16 hours. The resulting cloudy solution was added 10 mL DCM, then filtered through dried Celite, and concentrated in vacuo. The title compound was obtained as gray foam (1.33 g, 90%).

Preparation of phosphinyl imine 2 and borylation product 3

*Imine **2a***:

To a 10 mL Schlenk tube with a Teflon-coated magnetic stirbar was added anhydrous $MgSO_4$ (0.62 g, 7 equiv) and 4 Å molecular sieves (2.0 g). The tube was put into a 250 °C oven for 24 h before dried in high vacuum when hot. After the tube was cooled down completely, (*S*,*S*)-phosphinamide **1** (200 mg, 0.74 mmol) was

[1] Prepared according to literature: Herrmann, W. A.; Kocher, C.; Gookn, L. J.; Artus, G. R. J. *Chem. Eur. J.* **1996**, *2*, 1627–1636.

[2] Laitar, D. S.; Tsui, E. Y.; Sadighi, J. P. *J. Am. Chem. Soc.* **2006**, *128*, 11036–11037.

added. The mixture was protected with argon atmosphere. Isovaleraldehyde (2 equiv) and DCM (5 mL) were added with syringe then. The reaction mixture was stirred vigorously for 5 days. The resulting slurry was filtered on dried Celite and washed thoroughly by DCM for 5 times. The filtrate was cocentrated at room temperature and then put into high vacuum overnight. The crude product was checked by ^{31}P NMR and ^{1}H NMR and used directly for next step without further purification. (*S*,*S*)-**2a**: colorless oil. ^{1}H NMR ($CDCl_3$, 400 MHz) δ 0.58 (CH_3, d, *J* = 6.7 Hz, 3H), 0.65 (CH_3, d, *J* = 6.7 Hz, 3H), 1.63–1.74 (CH, m, 1H), 1.92–1.98 (CH_2, m, 1H), 2.02–2.08 (CH_2, m, 1H), 2.18–2.63 (CH_2, m, 4H), 3.36–3.54 (CH, m, 2H), 7.07–7.28 (Ar-H, m, 10H), 8.14–8.25 (N=CH, m, 1H). ^{31}P NMR ($CDCl_3$, 162 MHz) δ 55.4 (s). ^{13}C NMR ($CDCl_3$, 100 MHz) δ 183.9, 183.8, 136.7, 136.7, 135.8, 135.7, 129.0, 128.9, 128.6, 128.4, 128.4, 127.9, 127.9, 126.9, 126.9, 126.7, 126.7, 48.8, 48.0, 47.9, 47.7, 45.5, 44.7, 32.4, 32.3, 27.8, 27.7, 25.5, 22.4, 22.3.

*General procedure to imine **2b**–**2d***:

To a 10 mL Schlenk tube with a Teflon-coated magnetic stirbar was added (*S*,*S*)-phosphinamide **1** (200 mg, 0.74 mmol). 5 mL of toluene was added then under argon atmosphere. Aldehyde (2 equiv) and $Ti(OiPr)_4$ (3.0 equiv) were added subsequently. The suspension was stirred at room temperature for 2–3 days. The reaction was monitored by ^{31}P NMR. When complete, the clear solution was concentrated in vacuo. The residue was purified by rapid silica gel column chromatography (silica gel was dried in 250 ^{o}C. Solvents were dried over molecular sieves. EtOAc/hexanes/NEt_3 = 1/1/0.02) to afford the hemiaminal **7** or its mixture with imine. The product was re-dissolved in $CDCl_3$ and monitored by ^{31}P NMR. After 3 hours, the solution was concentrated again to afford the imine as the main product.

Imine ***2b*** (mixture with hemiaminal, 10:1):

Colorless oil. ^{1}H NMR ($CDCl_3$, 400 MHz) δ 0.79 (CH_3, d, J = 6.9 Hz, 3H), 0.84 (CH_3, d, J = 6.9 Hz, 3H), 2.26–2.71 (m, 5H), 3.44–3.59 (PCH, m, 2H), 7.15–7.35 (Ar-H, m, 10H), 8.17 (N=CH, dd, J = 4.1 Hz, J = 33.4 Hz, 1H). ^{31}P NMR ($CDCl_3$, 162 MHz) δ 55.2 (s). [For hemiaminal, two isomers, δ 51.3 (s) and 49.8 (s)] ^{13}C NMR ($CDCl_3$, 100 MHz) δ 187.7, 187.5, 136.9, 136.8, 135.9, 135.9, 129.3, 129.2, 129.1, 129.0, 128.7, 128.4, 128.3, 128.1, 128.0, 127.0, 127.0, 126.7, 126.7, 48.9, 48.2, 45.5, 44.6, 37.0, 36.8, 32.6, 32.5, 27.8, 27.7, 25.4, 17.9, 17.8.

Imine ***2c*** (mixture with hemiaminal, ~2:1):

Colorless oil. 1H NMR ($CDCl_3$, 400 MHz) δ 0.88–1.64 (m, 11H), 2.03–2.71 (m, 5H), 3.43–3.59 (PCH, m, 2H), 7.16–7.42 (Ar-H, m, 10H), 8.15 (N=CH, dd, J = 4.2 Hz, J = 34.4 Hz, 1H). 31P NMR ($CDCl_3$, 162 MHz) δ 55.2 (s). [For hemiaminal, two isomers, δ 51.5 (s) and 50.0 (s)]

Imine ***2d*** (mixture with hemiaminal, ~3:1):

Colorless oil. ^{1}H NMR ($CDCl_3$, 400 MHz) δ 1.97–2.69 (m, 8H), 3.42–3.60 (PCH, m, 2H), 6.94–7.37 (Ar-H, m, 15H), 8.33 (N=CH, td, J = 3.4 Hz, J = 33.0 Hz, 1H). ^{31}P NMR ($CDCl_3$, 162 MHz) δ 55.5 (s). [For hemiaminal, two isomers, δ 52.0 (s) and 49.8 (s)]

General procedure to imine ***2e*** *and* ***2f***:

To a 10 mL Schlenk tube with a Teflon-coated magnetic stirbar was added (*S*,*S*)-phosphinamide **1** (200 mg, 0.74 mmol). 5 mL of toluene was added then under argon atmosphere. Aldehyde (1.1 equiv) and $Ti(OiPr)_4$ (2.0 equiv) were added subsequently. The suspension was stirred at 80 °C for 20 hours before cooled down

to room temperature. Water (7 equiv) was added then and the mixture was stirred vigorously for 3 minutes. The suspension was filtered by Celite and the filtrate was then concentrated in vacuo. The crude product was checked by ^{1}H NMR and ^{31}P NMR, and used for next step without further purification.

Imine ***2e*** (crude):

Pale yellow solid. 1H NMR ($CDCl_3$, 400 MHz) δ 2.36–2.76 (CH_2, m, 4H), 3.68–3.76 (PCH, m, 2H), 7.08–7.71 (Ar-H, m, 15H), 8.77 (N=CH, d, J = 32.6 Hz, 1H). ^{31}P NMR ($CDCl_3$, 162 MHz) δ 56.2 (s).

Imine ***2f*** (crude):

Pale yellow solid. 1H NMR ($CDCl_3$, 400 MHz) δ 2.32–2.74 (CH_2, m, 4H), 3.52–3.62 (PCH, m, 2H), 3.84 (OCH3, s, 3H), 6.87–6.91 (m, 2H), 6.99–7.66 (m, 12H), 8.48 (N=CH, d, J = 32.5 Hz, 1H). ^{31}P NMR ($CDCl_3$, 162 MHz) δ 56.3 (s).

General procedure for the borylation reaction and GAP operation

A 10 mL Schlenk tube was charged with the crude imine **2**, B_2Pin_2 (375 mg, 2 equiv), catalyst ICyCuO*t*-Bu (54 mg, 20 mol %) and 4 Å Molecule Sieves (2.0 g). The mixture was protected with argon atmosphere. Toluene (4 mL) was added with syringe then. The reaction mixture was stirred vigorously for 3 days. The resulting slurry was filtered on Celite and washed by EtOAc for 5 times. The filtrate was condensed and then checked by ^{31}P NMR to determine the conversion and the crude dr value. GAP operations: 1) Method A: The filtrate was re-dissolved in EtOAc and washed with 1 N HCl, water and brine successively, then dried over anhydrous Na_2SO_4. The crude product was obtained by filtration and concentration then. 5 mL of hexanes was added to triturate the crude product. After 30 minutes, the organic

extraction layer was decanted and another 5 mL hexanes was added again. The resulting slurry was then filtered and the solid was washed with 2 mL hexanes. The solid product was collected and dried in vacuo. The yield was calculated and the dr value and purity were checked by ^{31}P NMR and ^{1}H NMR. 2) Method B: The filtrate was re-dissolved in EtOAc/hexanes (20 mL, v/v = 1:1), and then filtered on Celite. The filtrate was concentrated and triturated with hexanes (5 mL). After 30 minutes, the organic extraction layer was decanted and another 5 mL hexanes was added again. The resulting slurry was then filtered and the solid was washed with 2 mL hexanes. The solid product was collected and dried in vacuo. The yield was calculated and the dr value and purity were checked by ^{31}P NMR and ^{1}H NMR.

(*S*,*S*,*R*)-**3a**:

dr > 99:1. White sold (56%). $[\alpha]^{25}_{,D}$ –127.1 (*c* 1.40, CH_2Cl_2). ^{1}H NMR ($CDCl_3$, 400 MHz) δ 0.58 (CH_3, d, *J* = 6.4 Hz, 3H), 0.59 (CH_3, d, *J* = 6.4 Hz, 3H), 0.92–0.97 (CH, m , 1H), 1.03–1.08 (CH_2, m , 1H), 1.12 (CH_3, s, 6H), 1.16 (CH_3, s, 6H), 1.21–1.28 (CH, m, 1H), 1.91–2.03 (CH_2, NH, m, 2H), 2.14–2.25(CH_2, m, 1H), 2.33–2.49 (CH_2, m, 2H), 2.62–2.72 (CH, m, 1H), 3.03–3.11 (CH, m, 1H), 3.41–3.51 (NCH, m, 1H), 7.19–7.41 (Ar-H, m , 10H). ^{31}P NMR ($CDCl_3$, 162 MHz) δ 53.3 (s). [For the (*S*, *S*, *S*)-**3a**, δ = 54.0 (s)] ^{13}C NMR ($CDCl_3$, 100 MHz) δ 137.3, 137.3, 137.2, 137.2, 128.9, 128.9, 128.7, 128.3, 127.9, 127.9, 126.7, 126.5, 83.7, 48.6, 47.8, 42.9, 31.6, 31.1, 31.0, 28.1, 28.0, 25.1, 24.7, 24.5, 22.7, 22.6, 22.3, 14.1. HRMS (ESI) m/z calcd for $C_{27}H_{40}BNO_3P$ ($[M + H]^+$): 468.2839; found: 468.2852.

(*S*,*S*,*R*)-**3b**:

dr > 99:1. White sold (26%). $[\alpha]^{25}_{,D}$ –13.8 (*c* 0.50, CH_2Cl_2). ^{1}H NMR ($CDCl_3$, 400 MHz) δ 0.53 (CH_3, d, *J* = 6.9 Hz, 3H), 0.57 (CH_3, d, *J* = 7.3 Hz, 3H), 1.16 (CH_3, s,

6H), 1.18 (CH_3, s, 6H), 1.30–1.38 (Me_2CH, m, 1H), 1.99–2.10 (m, 1H), 2.18–2.29 (m, 2H), 2.34–2.50 (m, 2H), 2.60–2.66 (m, 1H), 3.01–3.10 (m, 1H), 3.45–3.55 (m, 1H), 7.19–7.39 (Ar-H, m, 10H). ^{31}P NMR ($CDCl_3$, 162 MHz) δ 52.2 (s). [For the (*S*, *S*, *S*)-**3b**, δ = 53.2 (s)] ^{13}C NMR ($CDCl_3$, 100 MHz) δ 137.5, 137.5, 128.9, 128.9, 128.8, 128.5, 128.1, 128.0, 126.7, 126.7, 126.6, 83.9, 77.3, 48.6, 47.9, 47.3, 46.5, 32.2, 31.0, 30.9, 28.2, 28.1, 25.0, 24.8, 19.5, 19.0. HRMS (ESI) m/z calcd for $C_{26}H_{38}BNO_3P$ ($[M + H]^+$): 454.2682; found: 454.2672.

(*S*,*S*,*R*)-**3c**:

dr = 99:1. White sold (40%). $[\alpha]^{25}_{,D}$ –31.9 (*c* 1.70, CH_2Cl_2). ^{1}H NMR ($CDCl_3$, 400 MHz) δ 0.50–0.68 (m, 2H), 0.80–0.93 (m, 3H), 1.15 (CH_3, s, 6H), 1.16 (CH_3, s, 6H), 1.59–2.63 (m, 12H), 2.97–3.05 (m, 1H), 3.42–3.52 (m, 1H), 7.17–7.37 (Ar-H, m, 10H). ^{31}P NMR ($CDCl_3$, 162 MHz) δ 52.8 (s). [For the (*S*, *S*, *S*)-**3c**, δ = 53.5 (s)] ^{13}C NMR ($CDCl_3$, 100 MHz) δ 137.8, 137.7, 137.4, 137.4, 128.9, 128.8, 128.7, 128.5, 128.1, 128.0, 126.7, 126.6, 120.1, 118.5, 83.9, 77.1, 60.4, 58.4, 48.6, 47.8, 47.6, 46.8, 42.0, 33.7, 33.3, 30.6, 30.5, 29.9, 29.7, 28.4, 28.3, 26.4, 26.3, 26.2, 25.2, 25.0, 25.0, 24.8, 24.7. HRMS (ESI) m/z calcd for $C_{29}H_{42}BNO_3P$ ($[M + H]^+$): 494.2995; found: 494.2982.

(*S*,*S*,*R*)-**3d**:

dr = 98:2. White sold (45%). $[\alpha]^{25}_{,D}$ –74.3 (*c* 0.40, CH_2Cl_2). ^{1}H NMR ($CDCl_3$, 400 MHz) δ1.15 (CH_3, s, 6H), 1.17 (CH_3, s, 6H), 1.40–1.48 (m, 2H), 1.97–2.06 (m, 1H), 2.19–2.49 (m, 6H), 2.71–2.79 (m, 1H), 3.00–3.08 (m, 1H), 3.45–3.55 (m, 1H), 6.94–6.96 (m, 2H), 7.08–7.12 (m, 1H), 7.16–7.39 (m, 12H). ^{31}P NMR ($CDCl_3$, 162 MHz) δ 53.2 (s). [For the (*S*, *S*, *S*)-**3c**, δ = 54.1 (s)] ^{13}C NMR ($CDCl_3$, 100 MHz) δ 142.3, 137.3, 129.0, 128.9, 128.9, 128.5, 128.2, 127.9, 127.9, 126.8, 126.7, 125.6, 84.1,

77.3, 48.6, 47.8, 47.0, 46.2, 35.9, 32.7, 31.0, 30.9, 28.0, 27.9, 25.0, 24.8. HRMS (ESI) m/z calcd for $C_{31}H_{40}BNO_3P$ ($[M + H]^+$): 516.2839; found: 516.2827.

(*S*,*S*,*R*)-**3e**:

dr > 99:1 after CC purification (51%). (*Not stable in air*) White sold. ^1H NMR ($CDCl_3$, 400 MHz) δ 0.94 (CH_3, s, 6H), 1.02 (CH_3, s, 6H), 2.07–2.41 (m, 4H), 2.78–2.85 (m, 1H), 3.11–3.18 (m, 1H), 3.49–3.57 (m, 1H), 3.94 (dd, *J* = 6.0 Hz, *J* = 11.9 Hz, 1H), 6.85–6.87 (m, 2H), 7.11–7.41 (m, 13 H). ^{31}P NMR ($CDCl_3$, 162 MHz) δ 52.8 (s). ^{13}C NMR ($CDCl_3$, 100 MHz) δ 141.9, 137.7, 137.6, 136.6, 136.5, 129.0, 129.0, 128.7, 128.6, 128.2, 127.9, 127.8, 127.0, 127.0, 126.4, 126.4, 84.4, 77.3, 48.2, 47.4, 46.2, 45.4, 32.6, 32.5, 27.2, 27.1, 24.6, 24.4. HRMS (ESI) m/z calcd for $C_{29}H_{36}BNO_3P$ ($[M + H]^+$): 488.2526; found: 488.2510.

(*S*,*S*,*R*)-**3f**:

dr > 99:1. White sold (44%). (*Not stable in air*) ^1H NMR ($CDCl_3$, 400 MHz) δ 0.94 (CH_3, s, 6H), 1.02 (CH_3, s, 6H), 2.06–2.39 (m, 4H), 2.76–2.85 (m, 1H), 3.08 (dd, *J* = 3.2 Hz, *J* = 9.2 Hz, 1H), 3.46–3.56 (m, 1H), 3.77 (OCH_3, s, 3H), 3.87 (dd, *J* = 5.5 Hz, *J* = 11.5 Hz, 1H), 6.75–7.39 (Ar-H, m, 14H). ^{31}P NMR ($CDCl_3$, 162 MHz) δ 52.8 (s). ^{13}C NMR ($CDCl_3$, 100 MHz) δ 158.3, 137.7, 137.7, 136.6, 136.6, 133.8, 129.3, 129.2, 129.1, 129.0, 128.8, 128.2, 127.9, 127.8, 127.0, 126.9, 126.3, 114.0, 84.4, 77.3, 55.3, 48.2, 47.4, 46.2, 45.4, 32.6, 32.5, 27.2, 27.1, 24.6, 24.4. HRMS (ESI) m/z calcd for $C_{30}H_{38}BNO_3P$ ($[M + H]^+$): 518.2632; found: 518.2609.

Deprotection of 3a

To a 50 mL Schlenk tube with a stirbar was added (*S*,*S*,*R*)-**3a** (200 mg, 0.43 mmol). The compound was protected with argon atmosphere. MeOH and H_2O were

added as a mixture (1:2, 11 mL) with syringe. Then, 1.5 equivalent of HCl (4.0 M in dioxane, 0.16 mL) was added dropwise at room temperature with a syringe. The suspension was stirred vigorously at room temperature for 16 h. 5 mL H_2O was added. The resulting slurry was then filtered. The solid was washed by 2 mL H_2O and dried in high vacuum to provide a pure and quantitive amount of (*R*,*R*)-phosphinic acid **5**. The combined filtrate was condensed to remove methanol, then extracted with 5 mL DCM. The water phase was separated and added 10 mL MeOH, then condensed under vacuum. A white solid was obtained then. DCM was added to extract the product and the pure (*R*)-3-methyl-1-(4',4',5',5'-tetramethyl-1',3',2'-dioxaborolan-2'-yl)butan-1-amine hydrogen chloride salt **4** (98 mg, 92%) was got after concentration. The ^{1}H NMR was in accord with that reported in Ellman's work. $[\alpha]^{25}_{,D}$ –7.4 (*c* 1.6, CH_2Cl_2). ^{1}H NMR ($CDCl_3$, 400 MHz) δ 0.97 (CH_3, d, *J* = 6.6 Hz, 6H), 1.30 (CH_3, s, 12H), 1.62–1.94 (CH, CH_2, m, 3H), 2.94 (NCH, br, 1H), 8.20 (NH_3, br, 3H).

NMR spectra

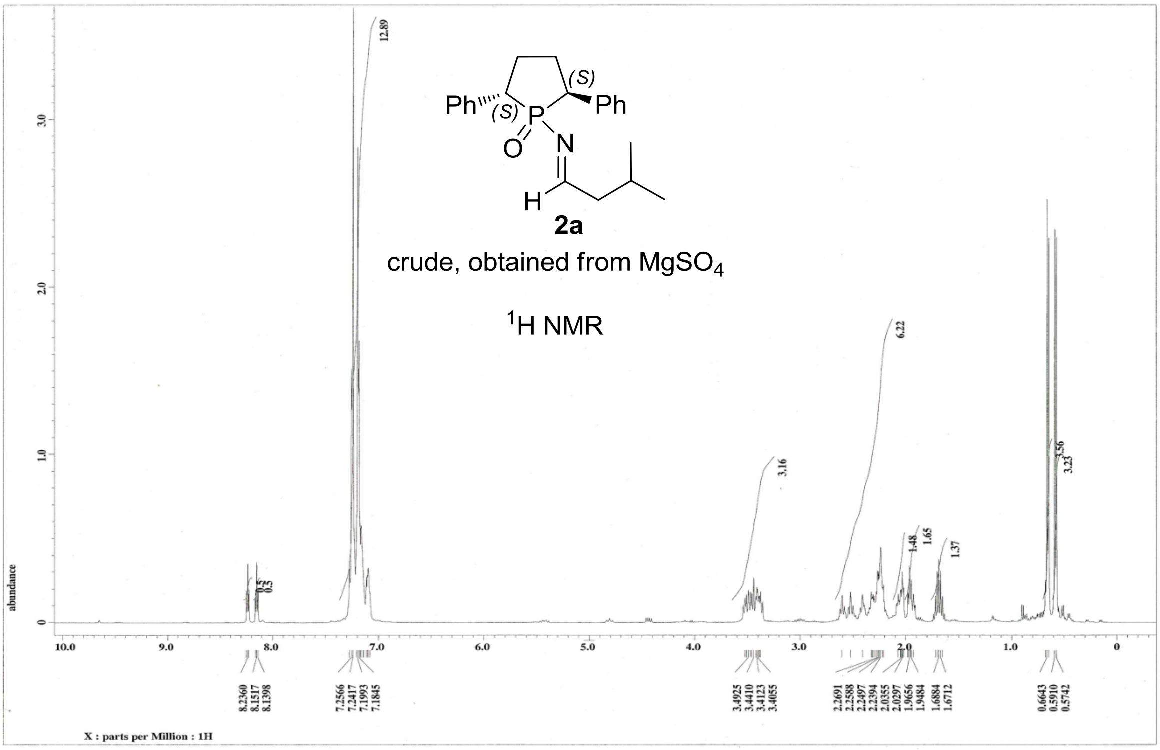

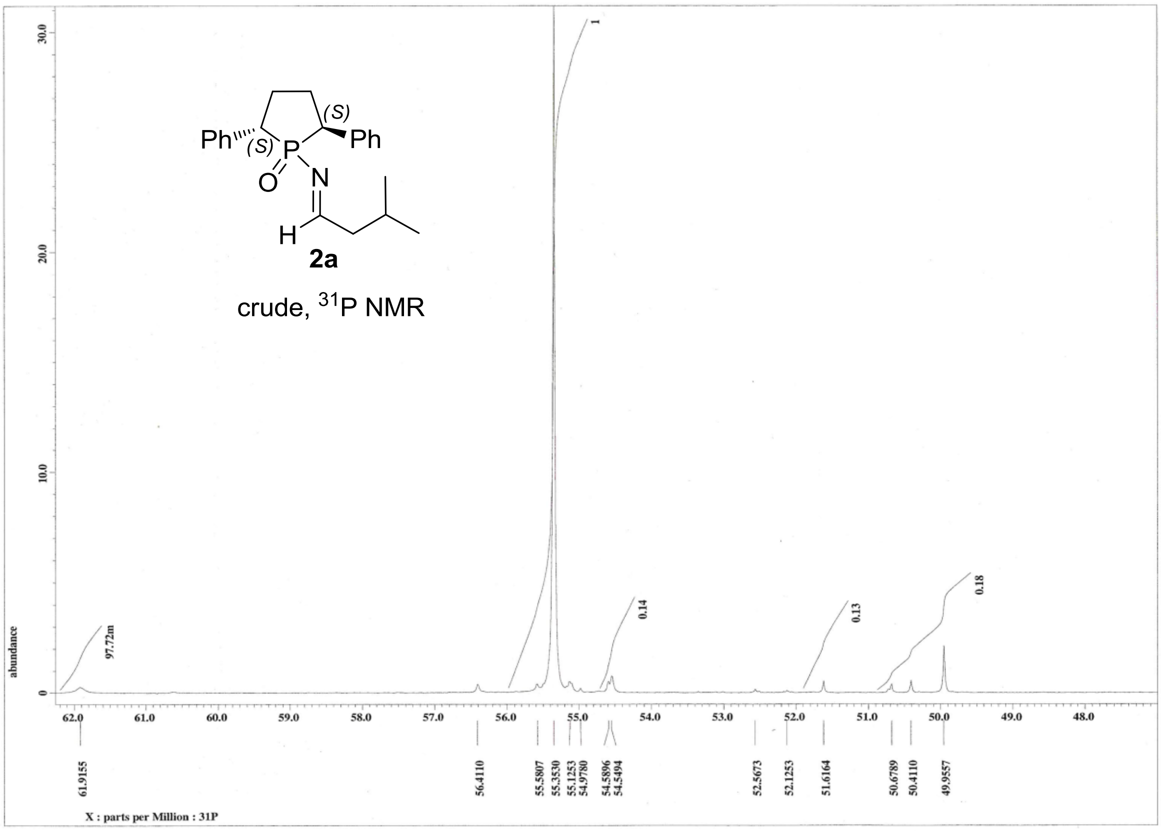
Ph
(S)
(S)
P
Ph
O
N
H
2a
crude, 31P NMR
abundance
X : parts per Million : 31P

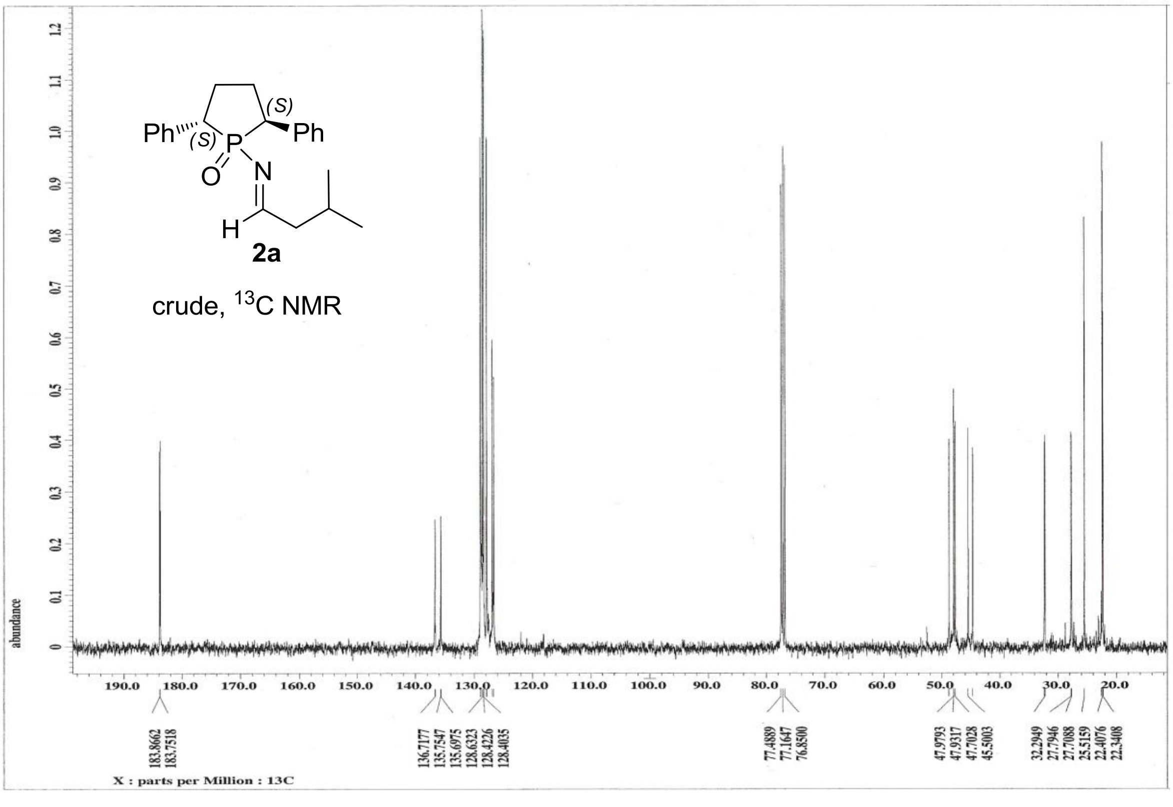
Ph
(S)
(S)
P
Ph
O
N
H
2a
crude, 13C NMR
abundance
X : parts per Million : 13C

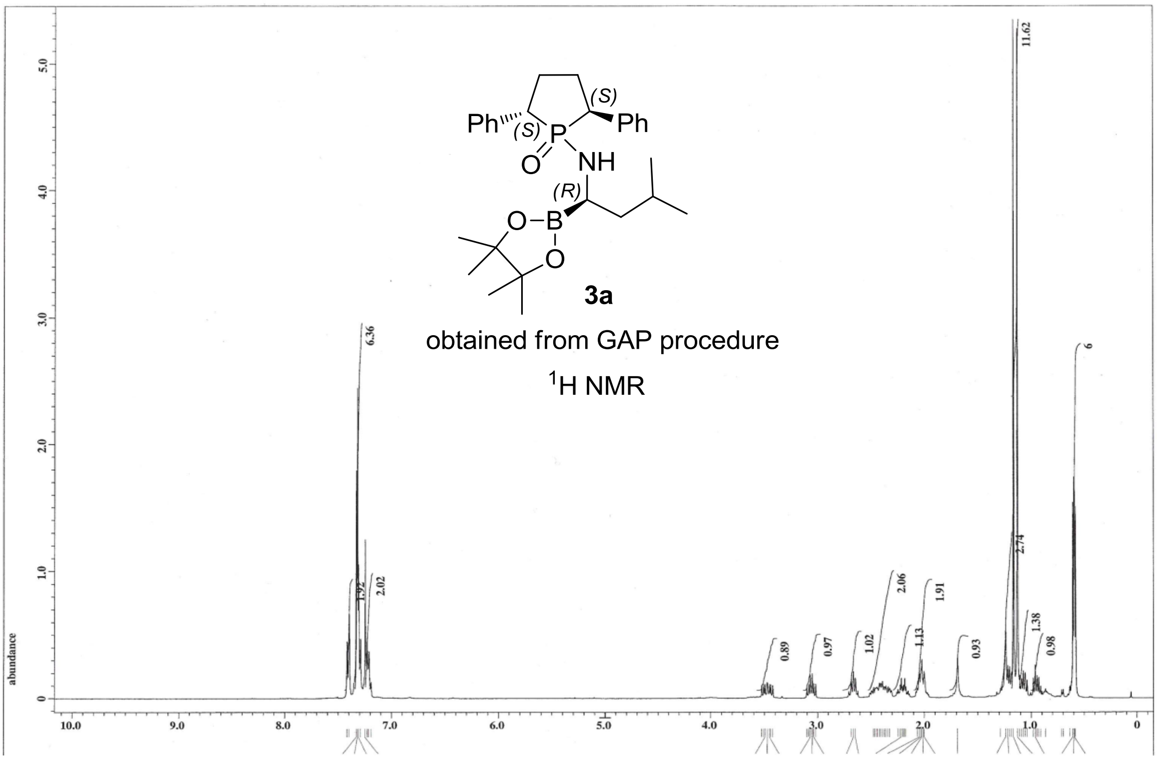
(S)
Ph
(S)
P
Ph
O
NH
(R)
O
B
O
3a
obtained from GAP procedure
1H NMR
6.36
1.92
2.02
0.89
0.97
1.02
2.06
1.13
1.91
0.93
11.62
2.74
1.38
0.98
6
abundance
X : parts per Million : 1H

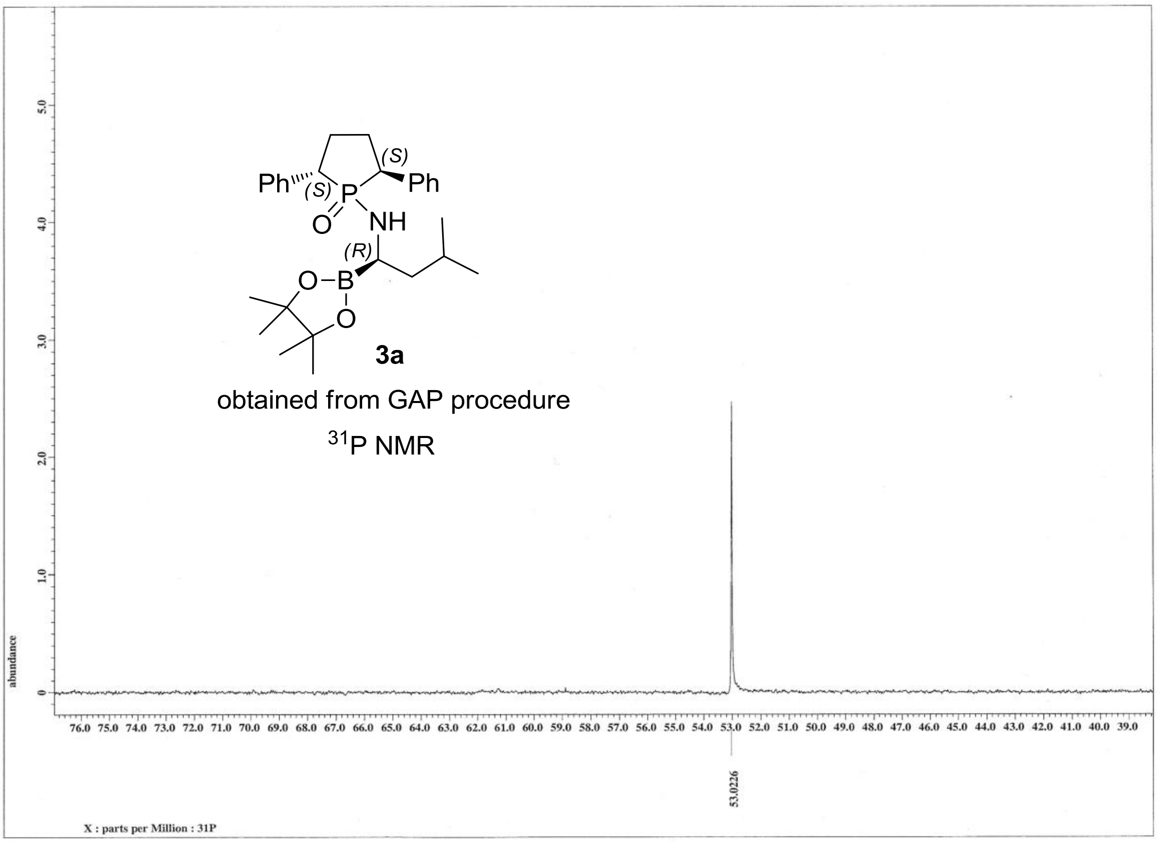
(S)
Ph
(S)
P
Ph
O
NH
(R)
O
B
O
3a
obtained from GAP procedure
31P NMR
abundance
53.0226
X : parts per Million : 31P

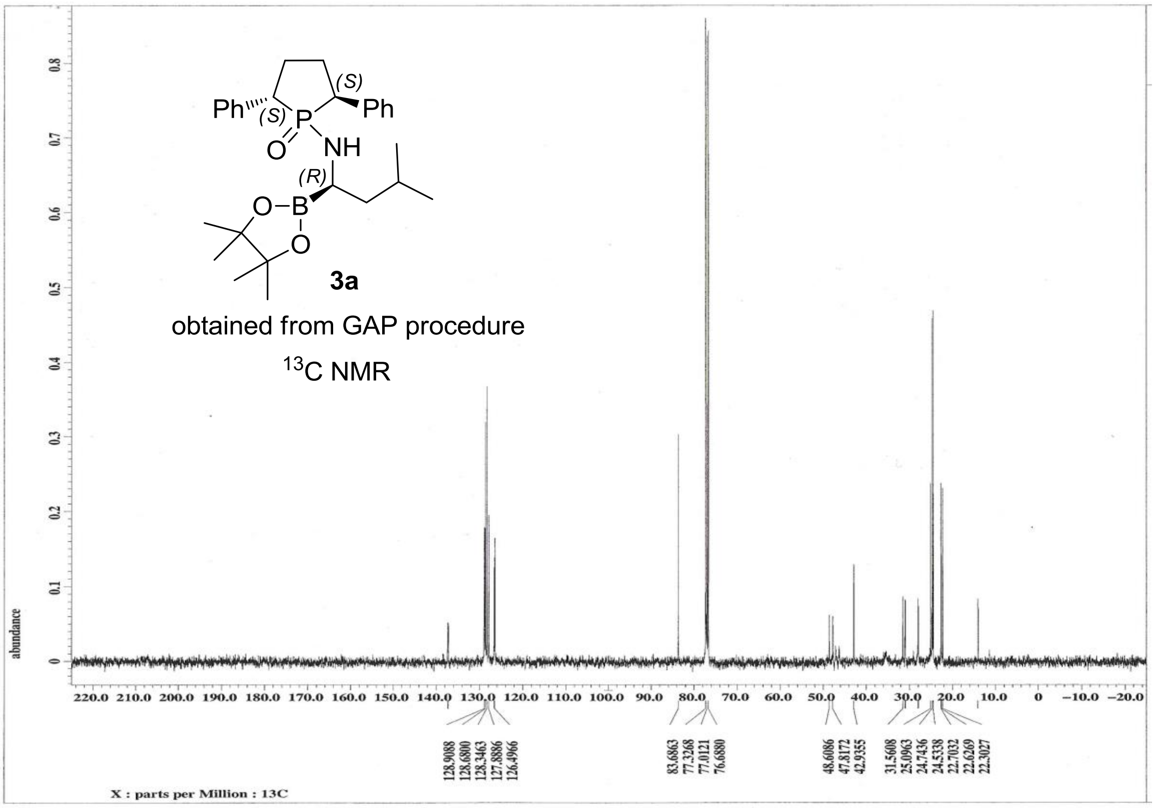
Ph
(S)
(S)
Ph
P
O
NH
(R)
O–B
O
3a
obtained from GAP procedure
13C NMR
abundance
X : parts per Million : 13C
128.9088
128.6800
128.3463
127.8886
126.4966
83.6863
77.3268
77.0121
76.6880
48.6086
47.8172
42.9355
31.5608
25.0963
24.7436
24.5338
22.7032
22.6269
22.3027

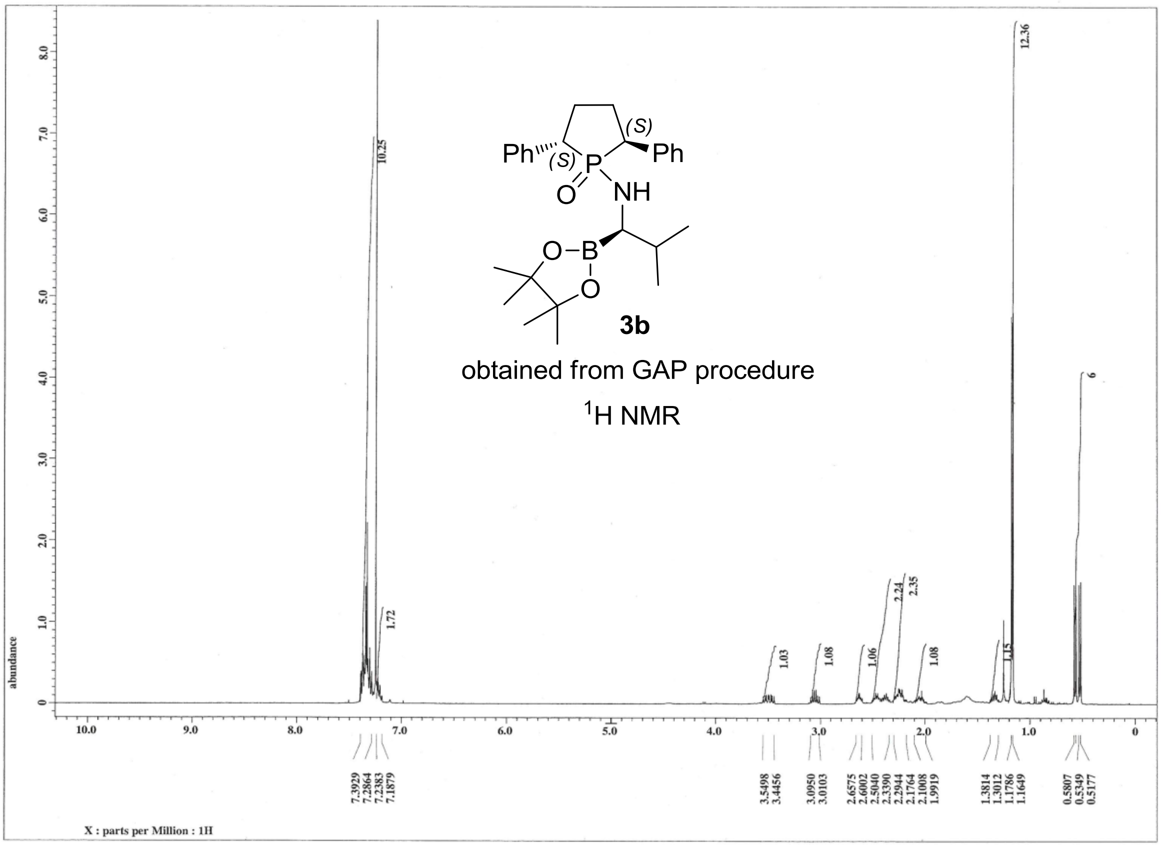
Ph
(S)
(S)
Ph
P
O
NH
O–B
O
3b
obtained from GAP procedure
1H NMR
abundance
X : parts per Million : 1H
10.25
1.72
1.03
1.08
1.06
2.24
2.35
1.08
1.15
12.36
6
7.3929
7.2864
7.2383
7.1879
3.5498
3.4456
3.0950
3.0103
2.6575
2.6002
2.5040
2.3390
2.2944
2.1764
2.1008
1.9919
1.3814
1.3012
1.1786
1.1649
0.5807
0.5349
0.5177

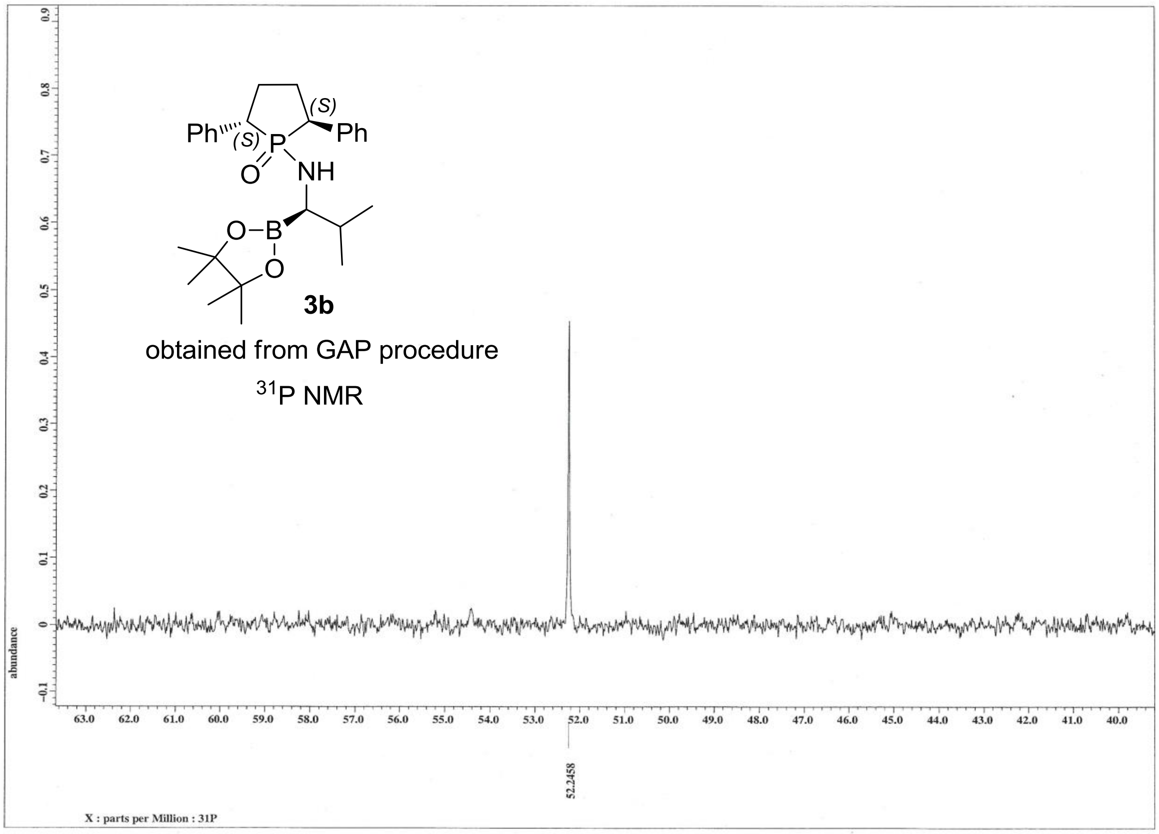
Ph
(S)
P
(S)
Ph
O
NH
O–B
O
3b
obtained from GAP procedure
31P NMR
abundance
X : parts per Million : 31P
52.2458

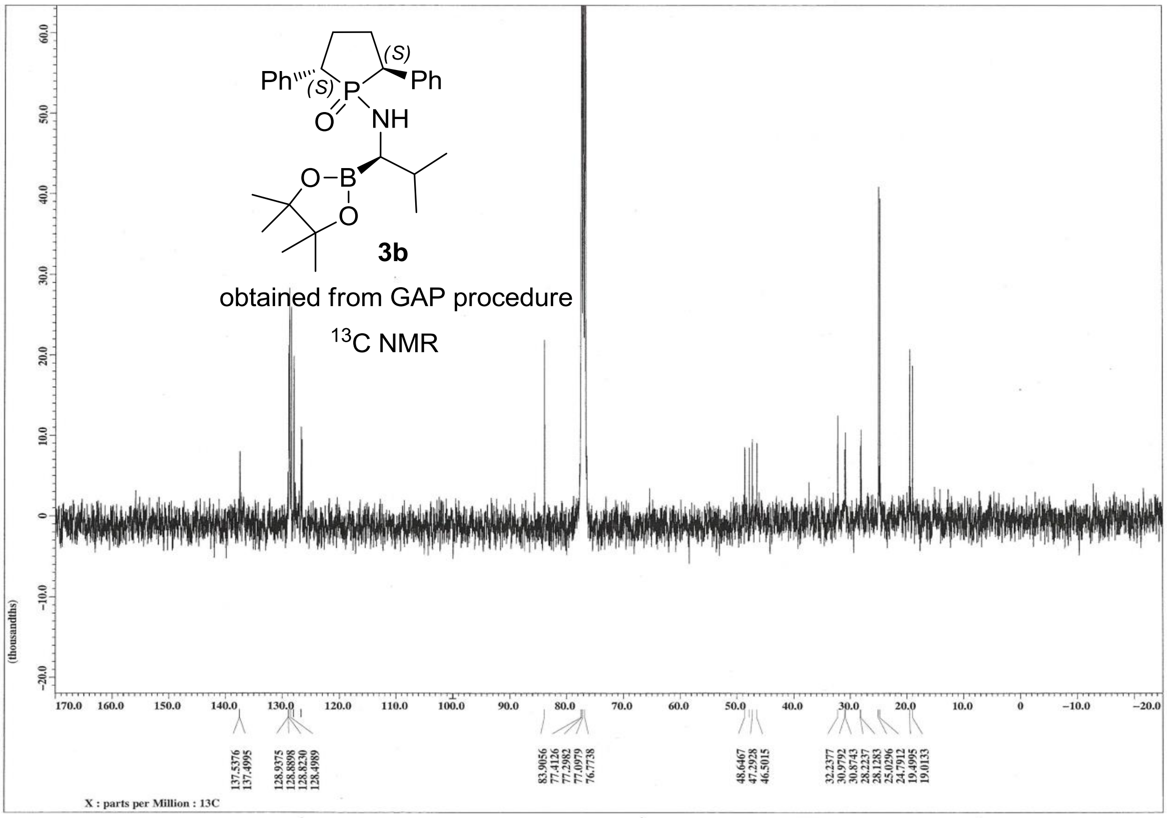
Ph
(S)
P
(S)
Ph
O
NH
O–B
O
3b
obtained from GAP procedure
13C NMR
(thousandths)
X : parts per Million : 13C
137.5376
137.4995
128.9375
128.8898
128.8230
128.4989
83.9056
77.4126
77.2982
77.0979
76.7738
48.6467
47.2928
46.5015
32.2377
30.9792
30.8743
28.2237
28.1283
25.0296
24.7912
19.4995
19.0133

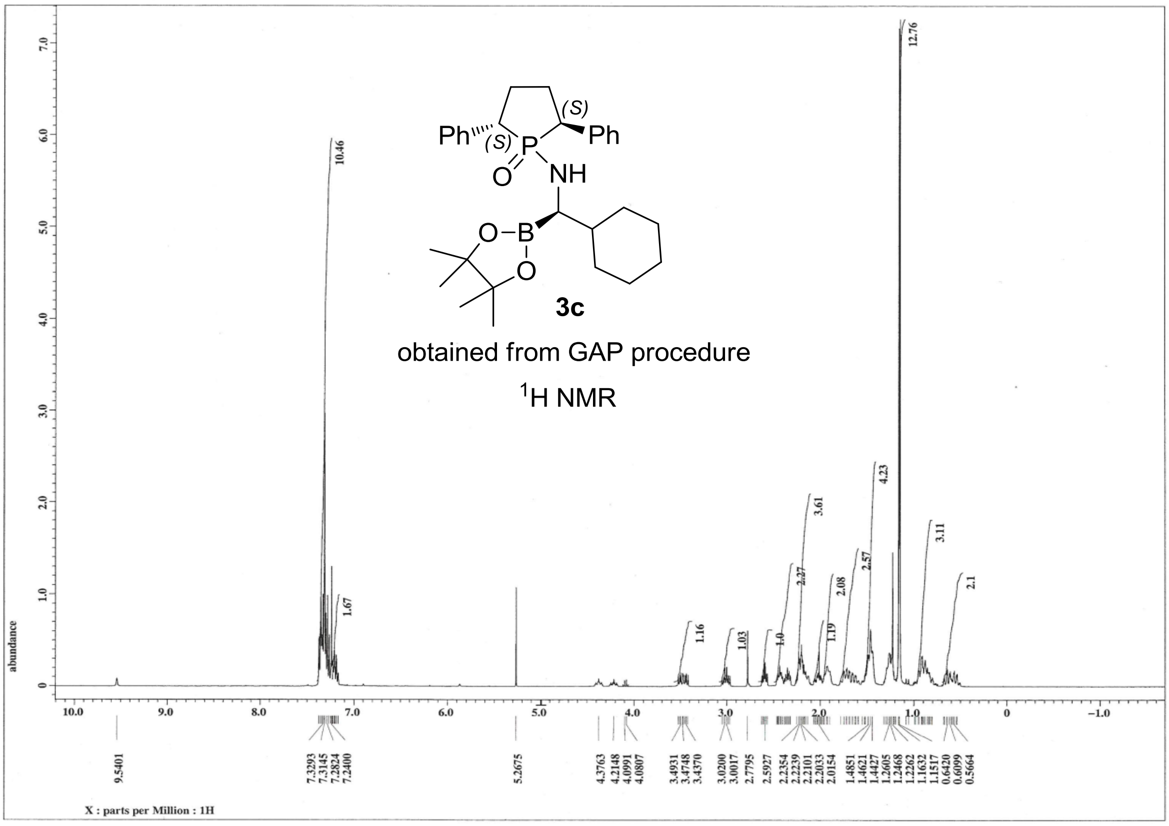
3c
obtained from GAP procedure
1H NMR
abundance
X : parts per Million : 1H

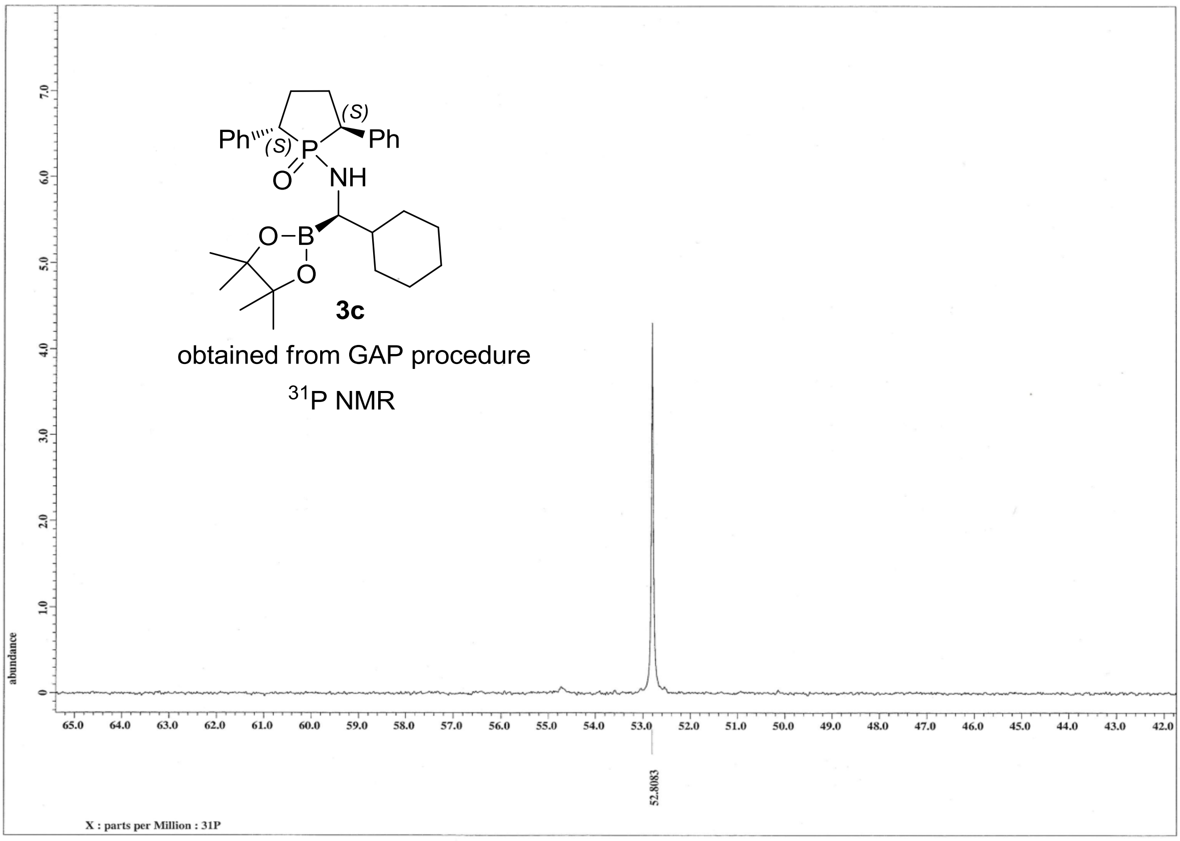
3c
obtained from GAP procedure
31P NMR
abundance
52.8083
X : parts per Million : 31P

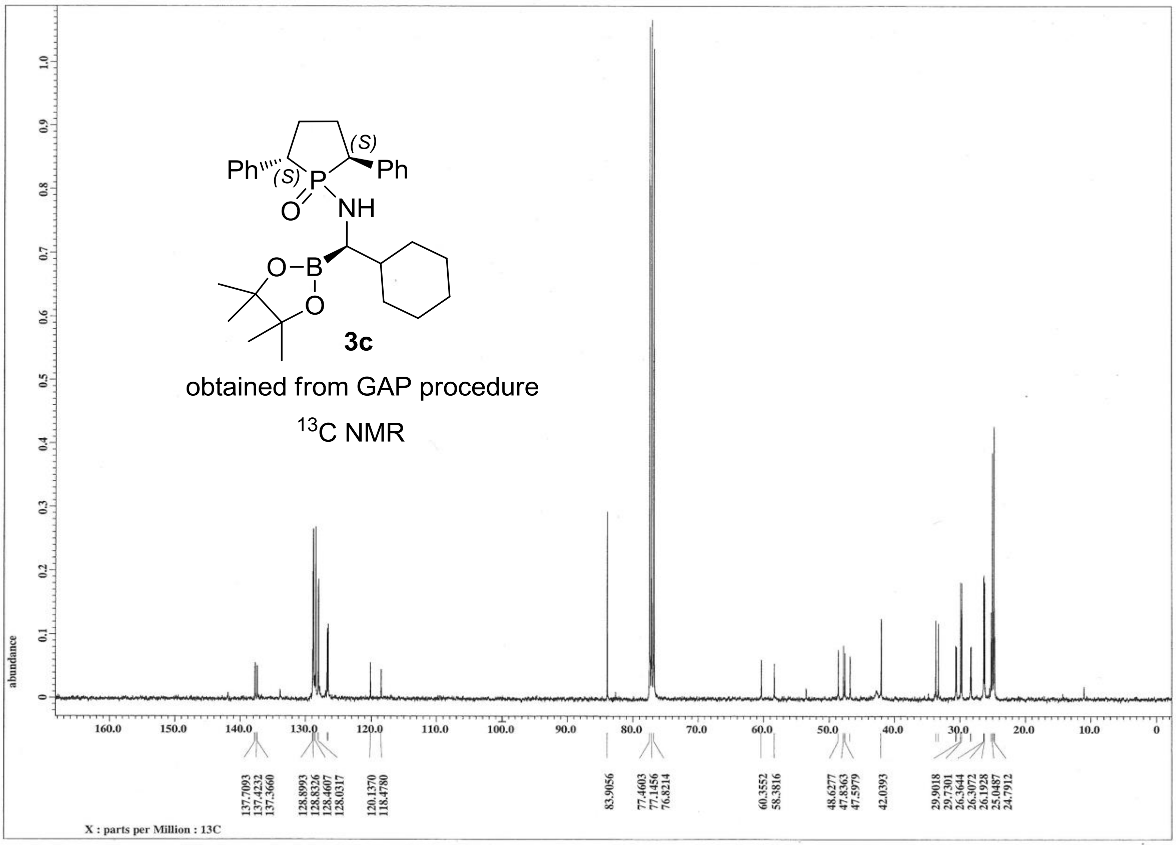
(S)
Ph
(S)
P
Ph
O
NH
O–B
O
3c
obtained from GAP procedure
13C NMR
abundance
X : parts per Million : 13C
137.7093
137.4232
137.3660
128.8993
128.8326
128.4607
128.0317
120.1370
118.4780
83.9056
77.4603
77.1456
76.8214
60.3552
58.3816
48.6277
47.8363
47.5979
42.0393
29.9018
29.7301
26.3644
26.3072
26.1928
25.0487
24.7912

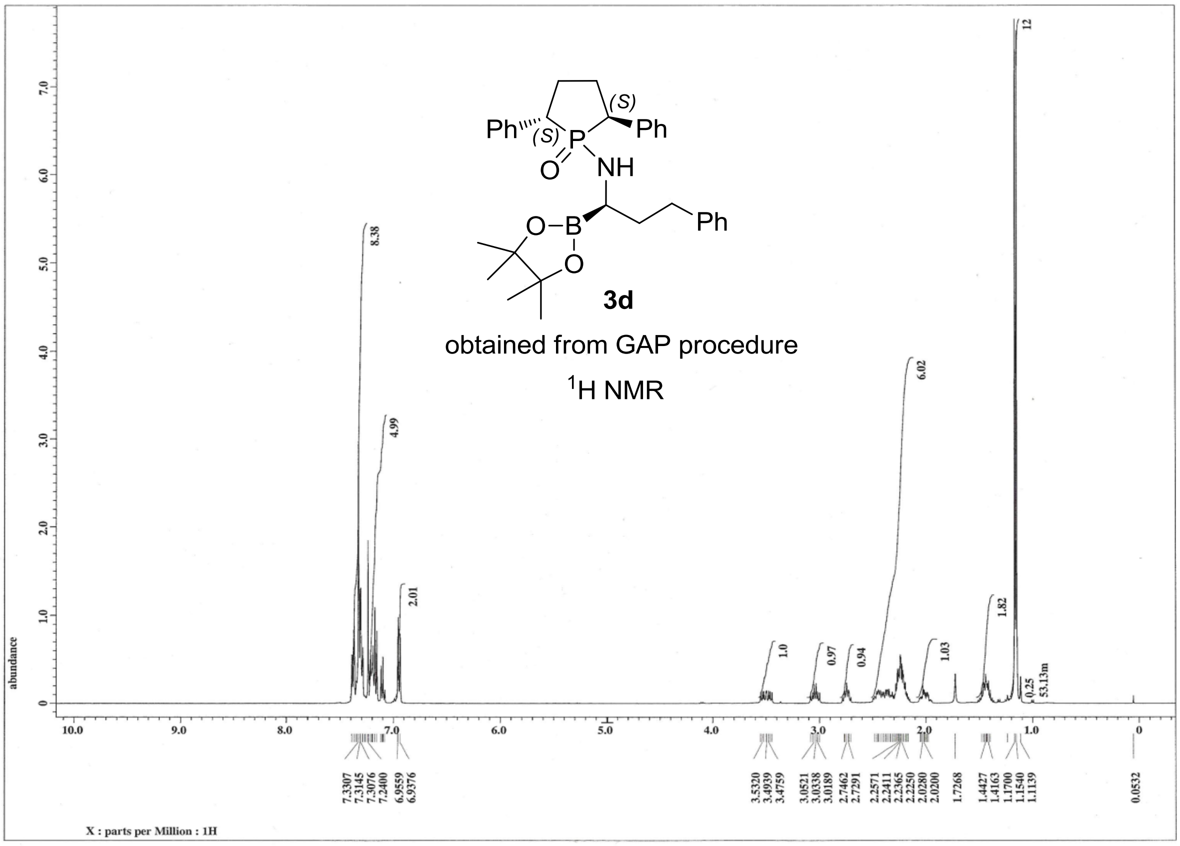
(S)
Ph
(S)
P
Ph
O
NH
O–B
O
Ph
3d
obtained from GAP procedure
1H NMR
abundance
X : parts per Million : 1H
8.38
4.99
2.01
1.0
0.97
0.94
6.02
1.03
1.82
12
0.25
53.13m
7.3307
7.3145
7.3076
7.2400
6.9559
6.9376
3.5320
3.4939
3.4759
3.0521
3.0338
3.0189
2.7462
2.7291
2.2571
2.2411
2.2365
2.2250
2.0280
2.0200
1.7268
1.4427
1.4163
1.1700
1.1540
1.1139
0.0532

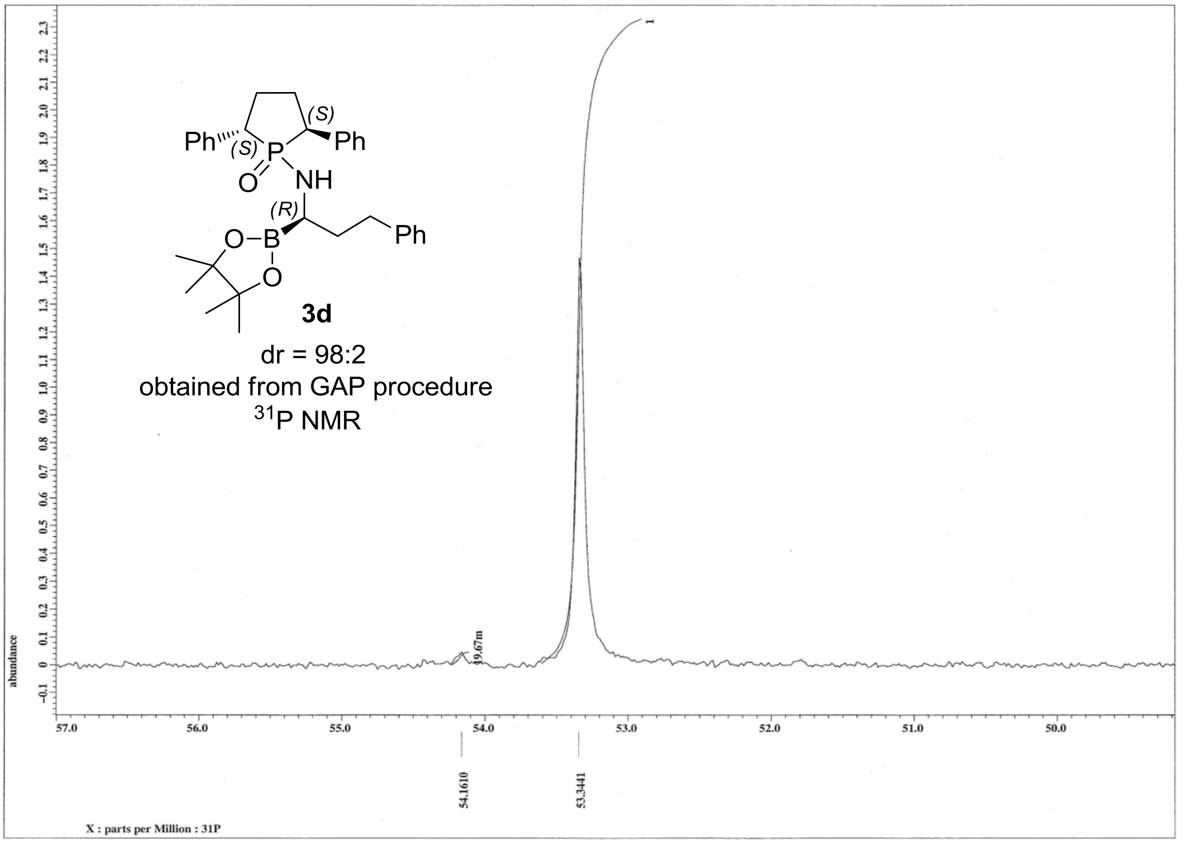
Ph
(S)
P
(S)
Ph
O
NH
(R)
O–B
O
Ph
3d
dr = 98:2
obtained from GAP procedure
^{31}P NMR
abundance
X : parts per Million : 31P
19.67m
54.1610
53.3441

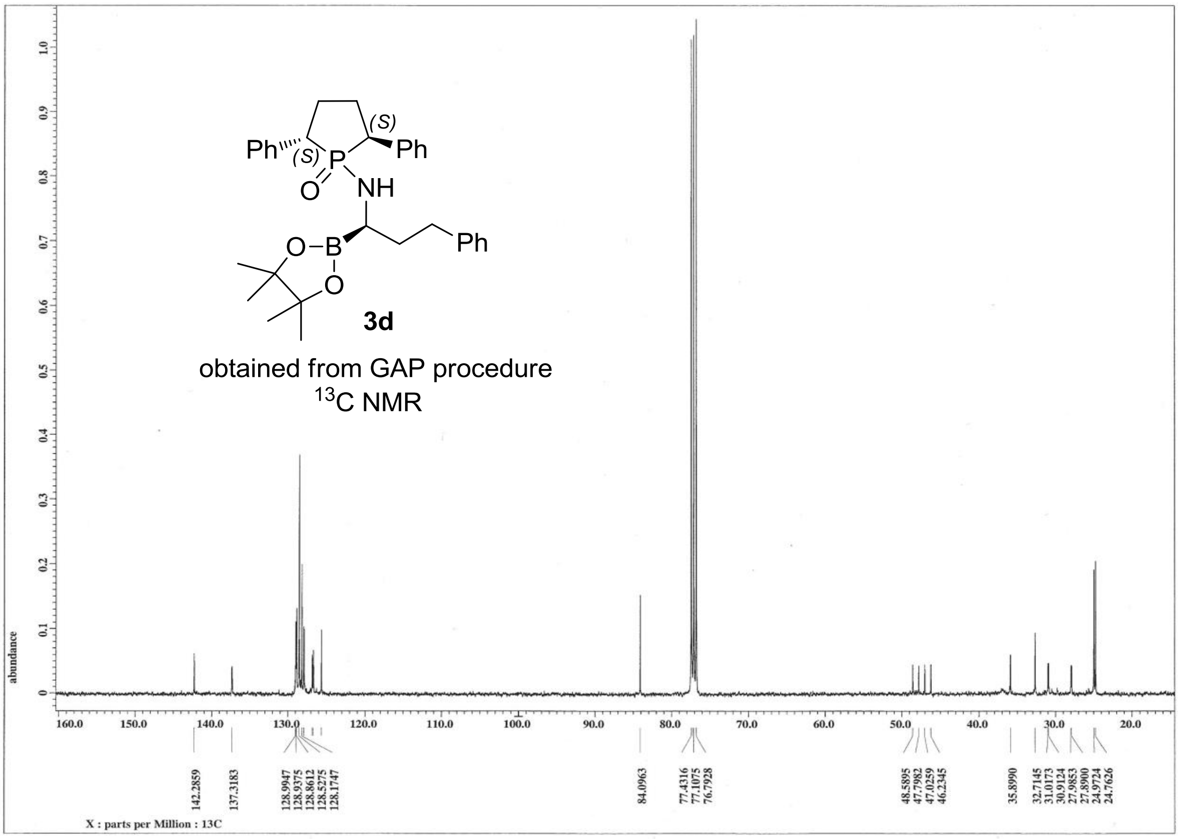
Ph
(S)
P
(S)
Ph
O
NH
O–B
O
Ph
3d
obtained from GAP procedure
^{13}C NMR
abundance
X : parts per Million : 13C
142.2859
137.3183
128.9947
128.9375
128.8612
128.5275
128.1747
84.0963
77.4316
77.1075
76.7928
48.5895
47.7982
47.0259
46.2345
35.8990
32.7145
31.0173
30.9124
27.9853
27.8900
24.9724
24.7626

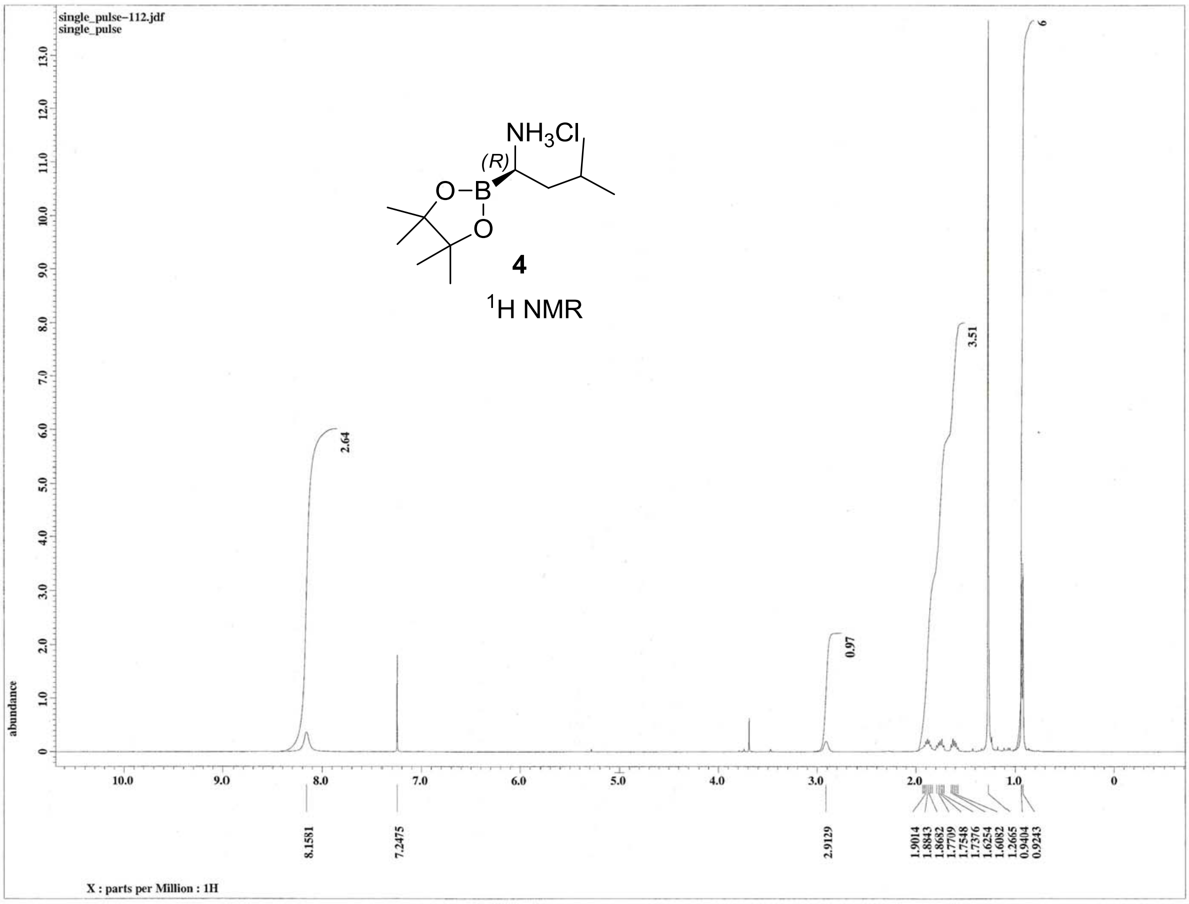
single_pulse-112.jdf
single_pulse
NH_3Cl
(R)
O
B
O
4
^{1}H NMR
abundance
X : parts per Million : 1H
2.64
0.97
3.51
6
8.1581
7.2475
2.9129
1.9014
1.8843
1.8682
1.7709
1.7548
1.7376
1.6254
1.6082
1.2665
0.9404
0.9243

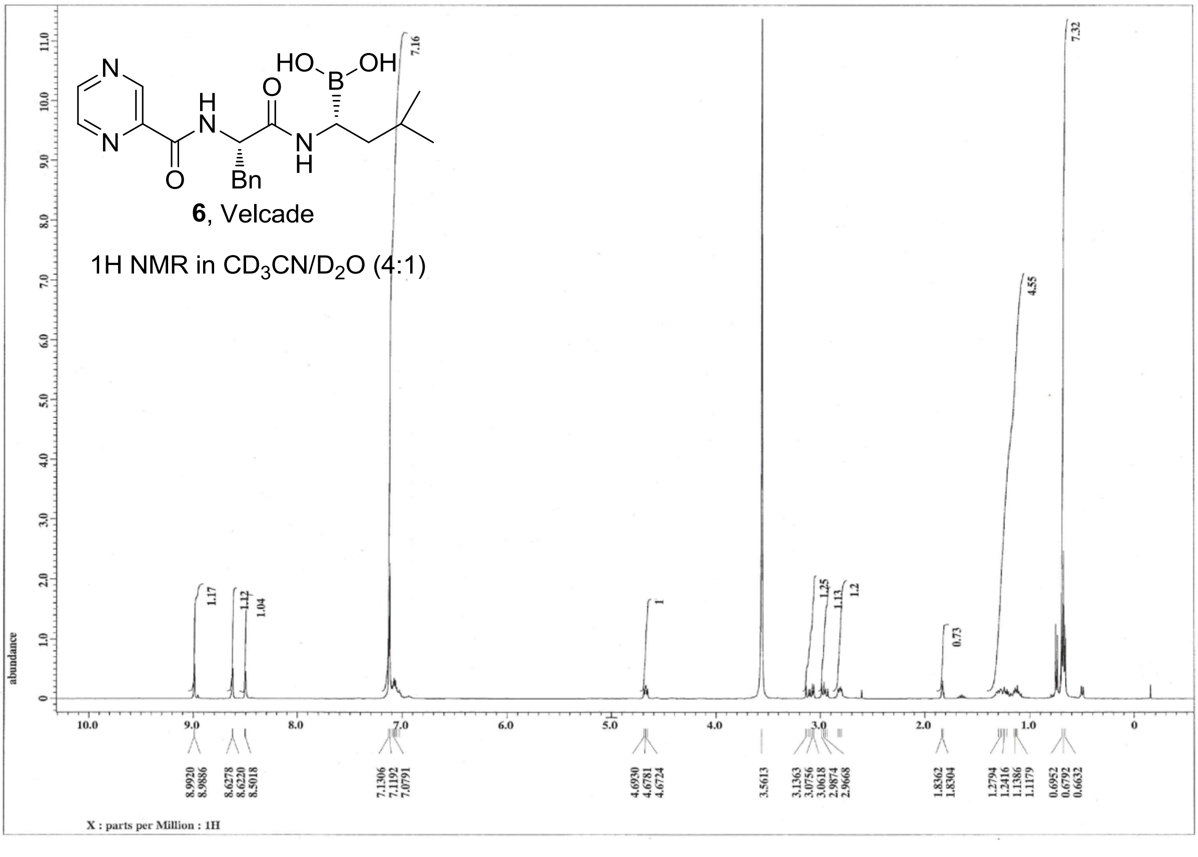
N
N
H
N
O
O
HO
B
OH
N
H
Bn
6, Velcade
1H NMR in CD_3CN/D_2O (4:1)
abundance
X : parts per Million : 1H
1.17
1.12
1.04
7.16
1
1.25
1.13
1.2
0.73
4.55
7.32
8.9920
8.9886
8.6278
8.6220
8.5018
7.1306
7.1192
7.0791
4.6930
4.6781
4.6724
3.5613
3.1363
3.0756
3.0618
2.9874
2.9668
1.8362
1.8304
1.2794
1.2416
1.1386
1.1179
0.6952
0.6792
0.6632
